# Genome-Wide RNA-Sequencing Reveals Massive Circular RNA Expression Changes of the Neurotransmission Genes in the Rat Brain after Ischemia–Reperfusion

**DOI:** 10.3390/genes12121870

**Published:** 2021-11-24

**Authors:** Ivan B. Filippenkov, Vasily V. Stavchansky, Alina E. Denisova, Liya V. Valieva, Julia A. Remizova, Ivan V. Mozgovoy, Elizaveta I. Zaytceva, Leonid V. Gubsky, Svetlana A. Limborska, Lyudmila V. Dergunova

**Affiliations:** 1 Institute of Molecular Genetics of National Research Center “Kurchatov Institute”, Kurchatov Sq. 2, 123182 Moscow, Russia; bacbac@yandex.ru (V.V.S.); lia97@mail.ru (L.V.V.); utoshkautoshka@gmail.com (J.A.R.); ivmstalker@gmail.com (I.V.M.); limbor@img.msk.ru (S.A.L.); lvd@img.msk.ru (L.V.D.); 2 Department of Neurology, Neurosurgery and Medical Genetics, Pirogov Russian National Research Medical University, Ostrovitianov str. 1, 117997 Moscow, Russia; dalina543@gmail.com (A.E.D.); gubskii@mail.ru (L.V.G.); 3 Faculty of Bioengineering and Bioinformatics, Lomonosov Moscow State University, Leninskie Gory, 119991 Moscow, Russia; batzaytceva@gmail.com; 4 Federal Center for the Brain and Neurotechnologies, Federal Biomedical Agency, Ostrovitianov str. 1, Building 10, 117997 Moscow, Russia

**Keywords:** tMCAO, RNA-Seq, circular RNA, mRNA, microRNA, neurotransmission

## Abstract

Ischemic brain stroke is one of the most serious and socially significant diseases. In addition to messenger RNAs (mRNAs), encoding protein, the study of regulatory RNAs in ischemic has exceptional importance for the development of new strategies for neuroprotection. Circular RNAs (circRNAs) have a closed structure, predominantly brain-specific expression, and remain highly promising targets of research. They can interact with microRNAs (miRNAs), diminish their activity and thereby inhibit miRNA-mediated repression of mRNA. Genome-wide RNA-Seq analysis of the subcortical structures of the rat brain containing an ischemic damage focus and penumbra area revealed 395 circRNAs changed their expression significantly at 24 h after transient middle cerebral artery occlusion model (tMCAO) conditions. Furthermore, functional annotation revealed their association with neuroactive signaling pathways. It was found that about a third of the differentially expressed circRNAs (DECs) originate from genes whose mRNA levels also changed at 24 h after tMCAO. The other DECs originate from genes encoding non-regulated mRNAs under tMCAO conditions. In addition, bioinformatic analysis predicted a circRNA–miRNA–mRNA network which was associated with the neurotransmission signaling regulation. Our results show that such circRNAs can persist as potential miRNA sponges for the protection of mRNAs of neurotransmitter genes. The results expanded our views about the neurotransmission regulation in the rat brain after ischemia–reperfusion with circRNA action.

## 1. Introduction

The problem of vascular diseases of the brain, and in particular of ischemic stroke, retains its medical and social significance due to the high rates of morbidity, disability, and mortality of this disease in the world [1,2]. A significant decrease or complete cessation of blood flow in brain can be caused by pathological narrowing of cerebral vessels, blockage by thrombus or embolus and other vascular events. Cerebral ischemia may also present manifestation of hematological diseases [3]. It is well known that cerebral ischemia causes a cascade of biochemical and transcriptome changes in brain tissues [4]. It has been shown that reperfusion after ischemia causes additional damage in brain cells, including the destruction of the brain microvascular endothelial cells, disturbance of the blood–brain barrier, accumulation of excess oxygen radicals, and activation of apoptosis [5,6]. The brain damage caused by ischemia–reperfusion (IR) leads to the disruption of the functioning of multiple genes [4,7,8]. To date, it has been shown that not only messenger RNAs (mRNAs) but also various types of regulatory RNAs are involved in the response to the pathological effects [9,10,11,12]. Of particular interest are circular RNAs (circRNAs), which have a covalently closed structure and are often formed in protein-coding genes during backsplicing [13,14]. CircRNAs demonstrate an increased resistance to the action of exonucleases and a predominantly brain-specific expression pattern [15,16,17].

Since 2016, the transcriptional profile of circRNAs under conditions simulating cerebral ischemia has been investigated. Recently, using microarray analysis in HT22 hippocampal cell culture under conditions of oxygen glucose deprivation-reoxygenation (OGD-R), which simulated damage after cerebral ischaemia and reperfusion, it was shown that circRNA expression is associated with the metabolic pathways of apoptosis and immune response [18]. Different authors have analyzed the differentially expressed circRNAs (DECs) of the transient middle cerebral artery occlusion model (tMCAO) in rodents using microarray analysis [19,20] and high-throughput circRNA sequencing (RNA-Seq) [21,22]. They showed that the DECs were associated with metabolic process, cell communication, and binding to proteins, ions, and nucleic acids [19], as well as with signalling pathways regulating the processes of cell survival and death [20]. In 2018, Han et al. and Bai et al. showed that circRNAs (*circHECTD1* and *circDLGAP4*, respectively) function as an microRNA (miRNA) sponge and adjust the size of infarct areas and blood-brain barrier damage activity [23,24]. Recently, additional evidences of the functioning of circRNAs as miRNA sponges in IR conditions were obtained by a number of scientific teams [25,26,27,28].

Previously, in a rat tMCAO model based on endovascular artery occlusion (90 min) and subsequent reperfusion, we revealed the activation of a large number of genes involved in inflammation, immune response, apoptosis, and stress response [29,30]. Simultaneously, a massive downregulation of genes that ensure the functioning of neurotransmitter systems was observed in tMCAO conditions. Additionally the brain expression profiling of key proteins involved in inflammation and cell death processes (MMP-9, c-Fos, and JNK), as well as neuroprotection and recovery (CREB) was revealed at 24 h after tMCAO [31]. In the present study, we used RNA-Seq to study the genome-wide response of the circRNA-transcriptome to the damaging effect of IR in the same tMCAO rat model conditions. We identified thousands of circRNAs, including about 400 DECs in the rat brain sub-cortex, which included necrotic and penumbra areas under the tMCAO model conditions. Functional annotation revealed that DECs predominantly associated with neuroactive signaling pathways. Comparison of results between high-throughput circRNA and mRNA sequencing was carried out. It was found that about a third of DECs originate from genes whose mRNA levels also changed at 24 h after tMCAO. The other DECs originate from genes encoding non-regulated mRNAs under tMCAO conditions. Bioinformatics analysis revealed a significant number of potential miRNA-circRNA and miRNA-mRNA interactions that are associated with the neurosignalling system of cells. The results reveal novel insight to the circRNAs as regulators of neurotransmission genetic response in rat brain cells after IR.

## 2. Materials and Methods

### 2.1. Animals

White 2-month-old male rats of the Wistar line (weight, 200–250 g) were obtained from the Experimental Radiology sector in A. Tsyb Medical Radiological Research Center, Obninsk, Russian Federation. The animals were divided into the “sham operation” (SH) and “ischemia–reperfusion” (IR) groups.

### 2.2. Transient Cerebral Ischemia Rat Model

Transient middle cerebral artery occlusion (tMCAO) was performed as previously described [29]. The rats were decapitated at 24 h after tMCAO (group “IR24”). The sham-operated rats (group “SH24”) were subjected to a similar surgical procedure under anaesthesia (neck incision and separation of the bifurcation), but without tMCAO. Each experimental group consisted of at least ten animals.

### 2.3. Magnetic Resonance Imaging

The magnetic resonance imaging (MRI) study of the characteristics of the ischemic injury of rat brains was carried out using small animal 7T ClinScan tomograph (Bruker BioSpin, Billerica, MA, USA). The standard protocol included the following modes: diffusion-weighted imaging (DWI) with mapping of the apparent diffusion coefficient (ADC) for assessing acute ischemic damage (TR/TE = 9000/33 ms; b factors = 0 and 1000 s/mm^2^; diffusion directions = 6; averages = 3; spectral fat saturation; FOV = 30 × 19.5 mm; slice thickness = 1.0 mm; matrix size = 86 × 56), and T2-weighted imaging (T2 WI) in the transverse plane (Turbo Spin Echo with restore magnetization pulse; turbo factor = 10; TR/TE = 5230/46 ms; averages = 2; spectral fat saturation; FOV = 30 × 21.1 mm; slice thickness = 0.7 mm; matrix size = 256 × 162). Three-dimensional time-of-flight magnetic resonance angiography (3D-TOF MRA) was used for visualization of the main arteries and control of the recanalization (3D Gradient Echo with RF spoiling and flow compensation; TR/TE = 30/4.55 ms; slabs = 4; flip angle = 70; averages = 1; FOV = 35 × 19.3 mm; slice thickness = 0.2 mm; matrix size = 320 × 176). For rats from the IR24 group, MRI was performed immediately before decapitation.

### 2.4. RNA Isolation

The ipsilateral subcortical structures were placed in RNAlater (Ambion, Austin, TX, USA) solution for 24 h at 4 °C and then stored at −70 °C. Total RNA was isolated using TRI Reagent (MRC, Cincinnati, OH, USA) and acid guanidinium thiocyanate-phenol-chloroform extraction [32]. The isolated RNA was treated with deoxyribonuclease I (DNase I) (Thermo Fisher Scientific Baltics UAB, Vilnius, Lithuania) in the presence of RiboLock ribonuclease (RNase) inhibitor (Thermo Fisher Scientific Baltics UAB, Vilnius, Lithuania), according to the manufacturer’s recommended protocol. Deproteinization was performed using a 1:1 phenol:chloroform mixture. The isolated RNA was precipitated with sodium acetate (3.0 M, pH 5.2) and ethanol. The RNA integrity was checked using capillary electrophoresis (Experion, BioRad, Hercules, CA, USA). RNA integrity number (RIN) was at least 9.0.

### 2.5. RNA-Seq

Total RNA isolated from the subcortical structures of the brain, including the lesion focus, was used in this experiment. The RNA-Seq experiment was conducted with the participation of ZAO Genoanalytika, Moscow, Russia. For RNA-Seq, the circRNA fraction of the total RNA was obtained using PureLink RNA Micro kit (Invitrogen, Thermo Fisher Scientific, Waltham, MA, USA) and “Trizol” (Invitrogen, Thermo Fisher Scientific, Waltham, Massachusetts, USA). The isolated RNA was treated with deoxyribonuclease I (DNase I) (Thermo Fisher Scientific, Waltham, MA, USA) in the presence of RiboLock ribonuclease (RNase) inhibitor (Thermo Fisher Scientific, Waltham, MA, USA), as well as ribosome RNA which was depleted using RiboMinus Eukaryote KIT (Ambion, A15017, Austin, TX, USA) according to the manufacturer’s recommended protocol. The obtained RNA was treated by RNAse R (Lucigen, RNR07250, Middleton, WI, USA) according to the manufacturer’s recommended protocol. cDNA (DNA complementary to RNA) libraries for circRNA were prepared using the NEBNext® Ultra™ II RNA Library Prep (New England Biolabs, E7770, Ipswich, MA USA). The concentration of cDNA libraries was measured using Qbit 2.0 and the Qubit dsDNA HS Assay Kit (Thermo Fisher Scientific, Waltham, MA, USA). The length distribution of library fragments was determined using the Agilent High Sensitivity DNA Kit (Agilent, Santa Clara, CA, USA).

For RNA-Seq, the miRNA fraction of the total RNA was obtained using PureLink RNA Micro kit (Invitrogen, Thermo Fisher Scientific, Waltham, MA, USA), “Trizol” (Invitrogen, Thermo Fisher Scientific, Waltham, MA, USA) and transcriptome plus protocol. cDNA libraries for miRNA were prepared using the NEBNext® Small RNA Library Prep Set for Illumina® (New England Biolabs, E7330, Ipswich, MA, USA). The quality of the cDNA libraries was checked using Bioanalyzer (Agilent, Santa Clara, CA, USA).

Sequencing was carried out using an Illumina HiSeq 2500 instrument. At least 20 million reads (1/200 nt) were generated for circRNAs and at least 5 million reads (1/50 nt) were generated for miRNAs.

### 2.6. RNA-Seq Data Analysis

Short sequence reads for circRNA were mapped onto the rat genome (rno5 and rno6 versions) using Bowtie2 (http://bowtie-bio.sourceforge.net/bowtie2/index.shtml) Version 2.3.4.2. The circRNA sequence was assembled using PTESFinder (http://sourceforge.net/projects/ptesfinder-v1/) [33]. All genes encoding circRNAs were annotated using the NCBI Reference Sequence databases [34,35]. The differential expression analysis of circRNAs in IR24 versus SH24 was carried out using the DESeq2 (https://bioconductor.org/packages/release/bioc/html/DESeq2.html). At least 3 animals were included in each comparison group. The levels of gene expression were measured as fragments per kilobase per million reads. Only genes that exhibited changes in expression > 1.5-fold and had a *P*-values adjusted using the Benjamini-Hochberg procedure lower 0.05 (*Padj* < 0.05) were considered.

### 2.7. Total RNA RNase R-Treatment

To carry out the synthesis of cDNA, 2 μg of DNAse free total RNA was treated with 10 μL of the reaction mixture and 1 U/μL of RNase R (Epicentre, New England Biolabs, Ipswich, MA, USA) in 1 × RNase R-buffer. The mixture was incubated at 37 °C for 1 h. Subsequently, 1 μL of 1 mM EDTA was added and used for the synthesis of cDNA.

### 2.8. cDNA Synthesis

cDNA synthesis was conducted in 20 μL of reaction mixture containing 2 mg of RNA using the reagents of a RevertAid First Strand cDNA Synthesis Kit (Thermo Fisher Scientific Baltics UAB, Vilnius, Lithunia) in accordance with the manufacturer’s instructions. Short random oligodeoxyribonucleotide primers were used to analyze circRNA. Oligo (dT)_18_ primers were used to analyze mRNA.

### 2.9. Reverse Transcription Polymerase Chain Reaction (RT–PCR)

To obtain samples for sequencing, 20 μL of the reaction mixture was prepared for reverse transcription polymerase chain reaction (RT-PCR), containing 2 μL of 0.2 × reverse transcriptase reaction sample, forward and reverse primers (5 pmol each), 0.2 mM of dNTP, 1 × High-Fidelity PCR Buffer with MgCl_2_, High-Fidelity PCR Enzyme Mix (5 U/μL) (Thermo Fisher Scientific, Waltham, MA, USA) and nuclease-free water (up to 20 μL). Specific primers were selected using the OLIGO Primer Analysis Software 6.31 (Molecular Biology Insights Inc., Colorado Springs, CO, USA) and were synthesized by the Evrogen Joint Stock Company, Moscow, Russia. Primers are presented in Supplementary Table S1. Amplification of cDNA was performed using a multichannel amplifier (DNA-Technology, Moscow, Russia) in the following mode: stage 1, 95 °C, 10 min; stage 2, 95 °C, 15 s; 65 °C, 25 s; 72 °C, 35 s (30 or 40 cycles).

### 2.10. Electrophoresis of PCR Products

PCR products were extracted with a mixture of chloroform and isoamyl alcohol (24:1). The DNA was dissolved in 9 μL of buffer (10 mM Tris (pH 9.0), 50 mM sodium chloride), 1 μL of 10 × dye (50 mM Tris (pH 8.27), 0.25% bromophenol blue, 60% glycerol) and applied to a horizontally positioned agarose gel (2% agarose, 1 × TAE, 0.6 μg/ml ethidium bromide). Electrophoresis was performed in 1 × TAE buffer for 35 min in a horizontal electrophoresis chamber (BioRad, Hercules, CA, USA) at 5 V/cm (Elf-8 power source, DNA-Technology, Moscow, Russia).

### 2.11. Sequencing of PCR Products

Individual bands were cut from the agarose gel. Isolation of DNA from the gel and sequencing of PCR products using the Sanger method were performed by the Evrogen Joint Stock Company, Moscow, Russia. Analysis of the Sanger sequencing results was conducted using Chromas Lite (Technelysium Pty Ltd, South Brisbane, Australia) software. The alignment of sequencing results with the sequence in the genome was performed using Basic Local Alignment Search Tool (https://blast.ncbi.nlm.nih.gov).

### 2.12. Real-Time RT–PCR

The 25 μL polymerase chain reaction (PCR) mixture contained 2 μL of 0.2 × reverse transcriptase reaction sample, forward and reverse primers (5 pmol each), 5 μL of 5 × reaction mixture (Evrogen Joint Stock Company, Moscow, Russia) including PCR buffer, Taq DNA polymerase, deoxyribonucleoside triphosphates (dNTP) and the intercalating dye SYBR Green I. Primers specific to the genes studied were selected using OLIGO Primer Analysis Software version 6.31 and were synthesized by the Evrogen Joint Stock Company (see Supplementary Table S1). The amplification of cDNAs was performed using a StepOnePlus Real-Time PCR System (Applied Biosystems, Foster City, CA, USA) in the following mode: stage 1 (denaturation), 95 °C, 10 min; stage 2 (amplification with fluorescence measured), 95 °C, 15 s; 65 °C, 25 s; 72 °C, 35 s (40 cycles).

### 2.13. Data Analysis of Real-time RT-PCR and Statistics

Two reference genes *Gapdh* and *Rpl3* were used to normalize the cDNA samples [36]. Calculations were performed using BestKeeper, version 1 [37] and Relative Expression Software Tool (REST) 2005 software (gene-quantification, Freising-Weihenstephan, Bavaria, Germany) [38]. The manual at the site ‘REST.-gene-quantification.info’ was used to evaluate expression target genes relative to the expression levels of the reference genes. The values were calculated as 2^Ct(ref)-Ct(tar)^, where Ct(tar) is the average threshold cycle (Ct) of the target gene, Ct(ref) is the average Ct of the reference gene. At least 5 animals were included in each comparison group. When comparing data groups, statistically significant differences were considered with the probability *P* < 0.05. Additional calculations were performed using Microsoft Excel (Microsoft Office 2010, Microsoft, Redmond, WA, USA).

### 2.14. Functional Analysis

Structural annotation of circRNAs and genome alignment was made using the UCSC Genome Browser (https://genome.ucsc.edu/) and NCBI databases (BLAST+ package, https://blast.ncbi.nlm.nih.gov/). gProfileR (https://biit.cs.ut.ee/gprofiler/gost) [39], DAVID version 6.8 (Database for Annotation, Visualization and Integrated Discovery, https://david.ncifcrf.gov/) [40] and The PANTHER (Protein ANalysis THrough Evolutionary Relationships, http://www.pantherdb.org/) [41] resources were used to annotate the functions of the differentially expressed circRNAs (DECs) and differentially expressed mRNAs (DEGs). When comparing data groups, statistically significant differences were considered with the probability *Padj* < 0.05. Hierarchical cluster analysis of DEGs and DECs was performed using Heatmapper (Wishart Research Group, University of Alberta, Ottawa, Canada) [42]. A volcano plot was constructed by Microsoft Excel (Microsoft Office 2010, Microsoft, Redmond, WA, USA). Primary search for orthologous DECs in human was carried out using circBase [43].

### 2.15. Construction of circ-miRNA-mRNA Network

The miRWalk2.0 (http://mirwalk.umm.uni-heidelberg.de/) [44] and TargetScan (http://www.targetscan.org/vert_80/) [45,46] resources were used to search for potential miRNA-mRNA pairs. Only those pairs that overlapped both miRWalk2.0 and TargetScan searching results were selected for analysis. Verified miRNA-mRNA pairs were analysed using miRTarBase (https://mirtarbase.cuhk.edu.cn/~miRTarBase/miRTarBase_2019/php/download.php) [47]. Potential miRNA-circRNA interactions were searched for using an integrated resource CircAtlas (http://159.226.67.237:8080/new/index.php) [48].

The Cytoscape 3.8.2 software (Institute for Systems Biology, Seattle, WA, USA) [49] was used to visualize the regulatory network. Additional calculations were performed using Microsoft Excel (Microsoft Office 2010, Microsoft, Redmond, WA, USA).

### 2.16. Availability of Data and Material

RNA sequencing data were deposited in the Sequence Read Archive database under accession code PRJNA523319 (SAMN10970974-SAMN10970979, https://www.ncbi.nlm.nih.gov/sra/PRJNA523319 accessed on 18 Febrary 2020) [50]. The annotated sequence was deposited into the GenBank with the accession number MK520929 [51].

## 3. Results

### 3.1. Magnetic Resonance Imaging (MRI)

**Figure 1:**
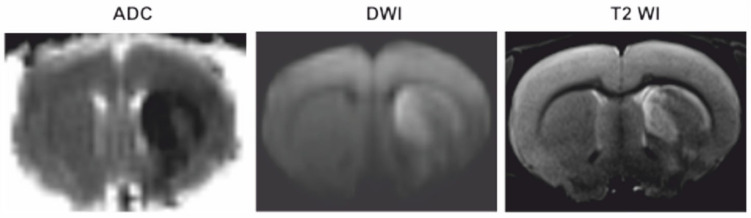
MRI of ischemic foci after tMCAO. DWI with an ADC map and T2 WI scans of the formation of ischemic injury areas with a subcortical localization in the brain of rats at 24 h after tMCAO.

Using the diffusion-weighted imaging (DWI) and T2-weighted imaging (T2 WI) modes of MRI, we detected the location and volume of ischemic foci in animals after tMCAO. A typical DWI with an ADC map and T2 WI scans of the formation of ischemic injury areas with a subcortical localization in the brain of rats at 24 h after tMCAO are shown in Figure 1.

### 3.2. RNA-Seq Analysis of circRNA Diversity in tMCAO Rat Model Conditions

Using RNA-Seq, we identified 11,106 circRNAs in the subcortical structures of the rat brain at 24 h after tMCAO. We assembled the full sequences of circRNAs which have the length of 521 (343; 816) nt (Me (LQ; UQ) – the median as well as the lower and upper (UQ) quartiles for the 25 and 75 percentile interval). Using the NCBI reference sequence database, we identified 3748 genes that encoded the assembled circRNAs in the subcortical structures of the rat brain at 24 h after tMCAO. Interestingly, there were 1661 genes, each of which encoded a single circRNA. There were also 143 genes that encoded more than 10 circRNAs (Supplementary Figure S1a). Among them were three genes (*Lrp1b*, *Arhgap10*, and *Rims2*) that encoded even more than 30 circRNAs.

**Figure 2:**
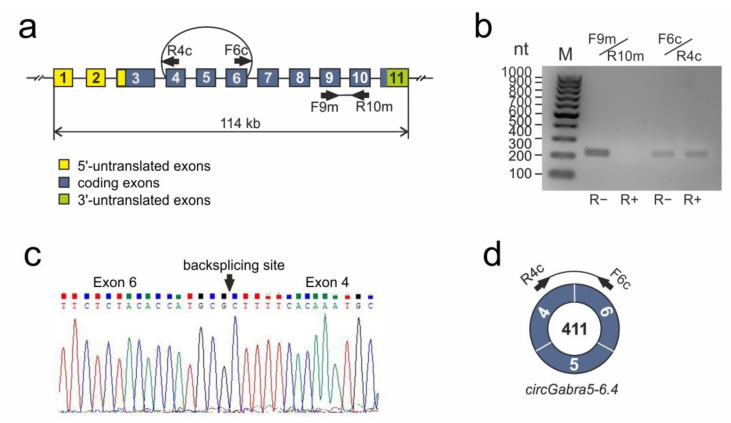
An analysis of circRNA diversity in tMCAO rat model conditions. (**a**) The exon-intron structure of the rat *Gabra5* gene. Exons were shown as numerated blocs and the separating introns, as straight lines connecting rectangles. Arcs connect the ends of exons involved in the formation of circRNA. Primers (F, forward; R, reverse) are shown as arrows, the direction indicates the 5′-3′-orientation. (**b**) Electrophoretic separation of the cDNA amplification products of the rat brain in the presence of primers complementary to exon sequences of the *Gabra5* gene. On the left, the position of markers bands is indicated by GeneRuler 100 bp DNA Ladder (Thermo Fisher Scientific, Waltham, MA, USA). (**c**) Sanger sequencing result of *circGabra5-6.4* matched to regions of exons that participated in back-splicing. It was visualized using Chromas Lite (Technelysium Pty Ltd, South Brisbane, Australia) software. (**d**) The structure of circRNA *circGabra5-6.4* of the *Gabra5* gene, whose formation was confirmed using PCR and Sanger sequencing. The number inside the circle is the length of circRNAs (nucleotides), figures in the circle segments are the numbers of exons inside the circRNAs.

Real-time reverse transcription polymerase chain reaction (RT−PCR) was used to verify the structure of the circRNA, for example, *circGabra5-6.4* for *Gabra5*. The total RNA sample from the subcortical structures of the rat brain was treated with RNase R (R+). The sample of total RNA fraction without RNase R (R−) treatment was used as a control. DNA complementary to RNA (cDNA) was amplified with outward-facing primers F6c/R4c matched to regions of exons that participated in back-splicing. cDNA amplification in the presence of inward-facing primers F9m/R10m complementary to the linear mRNA sequence of *Gabra5* was used as a control. The characterization of primers is shown in Figure 2a and in Supplementary Table S1. We showed that the level of RNA containing back-splicing exons 4 and 6 (primers F6c/R4c) was retained in the R+ fraction compared with the level in the R− fraction. Concomitantly, the level of RNA containing linear spliced exons 9 and 10 (primers F9m/R10m) decreased in the R+ fraction (Figure 2b). Additionally, the amplified fragments obtained using primers F6c/R4c were isolated from the gel, purified, and sequenced using the Sanger method.

It was possible to establish the back-splicing site of circRNA (*circGabra5-6.4*) of the *Gabra5* gene (Figure 2c). The GenBank database recorded the *circGabra5-6.4* detected by us as MK520929. Thus, results of the Sanger method verified the RNA-Seq results. Figure 2d is schematic representation of the detected circRNA for the *Gabra5* gene.

### 3.3. RNA-Seq Analysis of Differential Expression of circRNAs at 24 h after tMCAO

**Figure 3:**
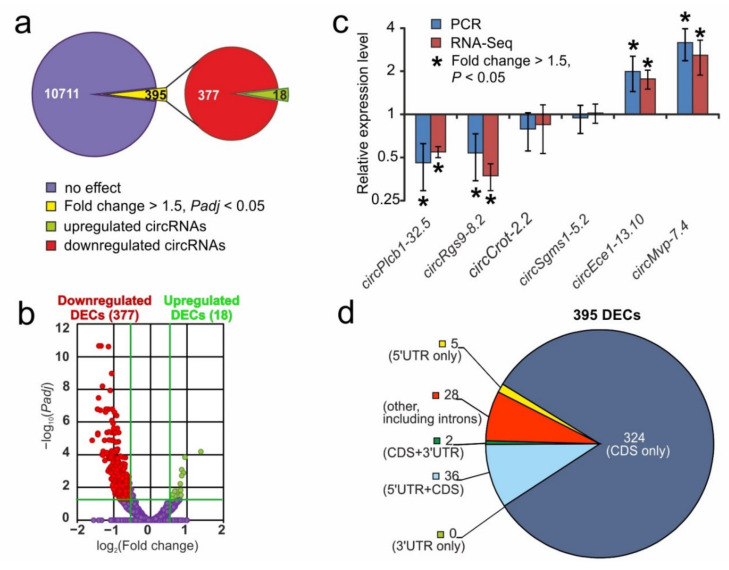
RNA-Seq analysis of DECs at 24 h after tMCAO. (**a**) RNA-Seq results in IR24 versus SH24. The numbers in the diagram sectors indicate the number of DECs. (**b**) A volcano plot shows the distributions of genes between the IS24 and IR24 groups. Up- and downregulated DECs are represented as red and green dots, respectively (fold change > 1.50. *Padj* < 0.05). Not differentially expressed circRNAs (non-DECs) are represented as dark purple dots (fold change ≤ 1.50. *Padj* ≥ 0.05). (**c**) RT-PCR verification of the RNA-Seq results. Data for comparison in IR24 versus SH24 are shown. Two reference mRNAs for *Gapdh* and *Rpl3* were used to normalize the PCR results. In each group, there were at least 5 rats. Four circRNAs that change the expression more than 1.5-fold from the baseline value and whose *p*-value was lower 0.05, as well as two other circRNAs, were selected for analysis. (**d**) Annotations of genomic regions mapping to DECs. Numbers in sectors indicate the number of DECs contained corresponding to structural elements of genome.

From 11,106 identified circRNAs, we revealed only 395 (18 up- and 377 downregulated) DECs in “ischemia-reperfusion” versus “sham operation” groups at 24 after tMCAO (IR24 versus SH24) (Figure 3a). A volcano plot (Figure 3b) illustrates the differences in circRNA expression between the IR24 and SH24 groups. The top five most highly upregulated DECs originated from *Mvp*, *Col4a1*, and *Cdyl*, while the top five most markedly downregulated DECs were from *Pde10a*, *Macf1*, *Mme*, and *Rgs9*.

RT–PCR analysis of the expression of two up- (*circEce1-13.10, circMvp-7.4*), two down- (*circPlcb1-32.5, circRgs9-8.2*) and two non-significantly (*circCrot-2.2, circSgms1-5.2*) regulated circRNAs was used to verify the RNA-Seq results. The characterization of primers is shown in Supplementary Table S1. The real-time RT–PCR results adequately confirmed the RNA-Seq data (Figure 3c).

The number of genes and DECs that were encoded by them are shown in Figure S1b (Supplementary Figure S1). So, the 395 DECs originated from 211 host genes. Interestingly, there were 137 genes, each of which encoded a single circRNA. There were also four genes that encoded more than seven circRNAs. Among them, there were *Lrp1b, Pde10a, Dgkb, and Prex2* genes that encoded 8, 9, 10 and 11 DECs, respectively. Using the UCSC Genome Browser, we annotated the structure of DECs at 24 h after tMCAO. We found that 82% of DECs contained coding exons (CDS) only, whereas 18% of DECs contained other structural elements of the genome, including untranslated regions (UTRs) (Figure 3d).

An additional pilot analysis using circBase revealed that at least 93 DECs may have orthologous human circRNAs. Among them, DECs related to *Birc6, Cacna1e, Grik2, Pde10a, Prex2, Prkcb, Robo2, Strn, Cct4, Fbxw4*, and other genes (Supplementary Table S2).

### 3.4. Comparative Analysis of Transcriptome Profile Changes of mRNAs and circRNAs at 24 h after tMCAO

**Figure 4:**
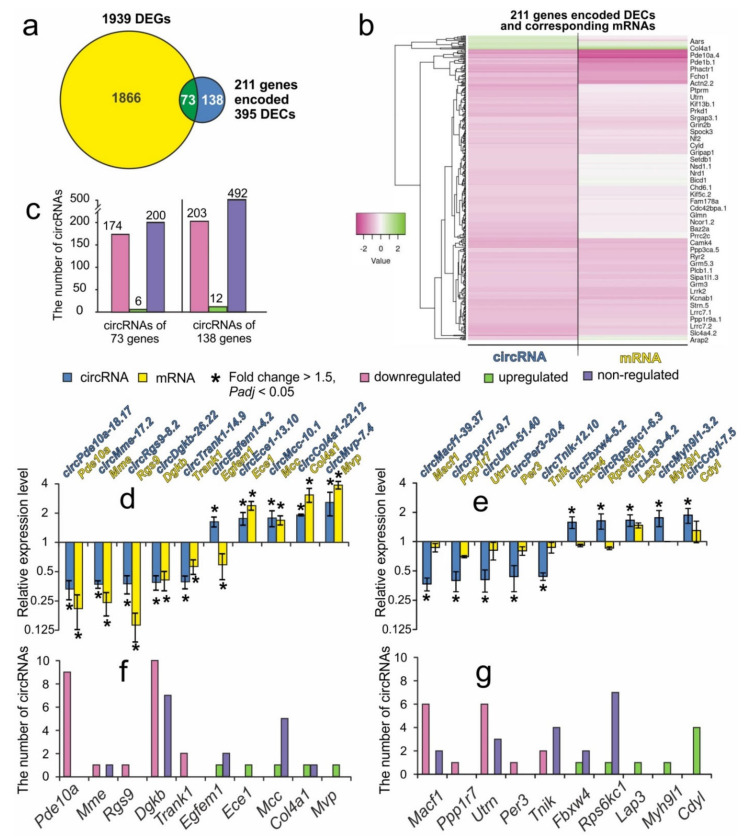
Comparative analysis of circRNAs and mRNAs expression changes at 24 h after tMCAO. (**a**) Schematic comparison of the transcriptome circRNA and mRNA profiles obtained in pairwise comparison IR24 versus SH24 by Venn diagram. The numbers in the diagram sectors indicate the number of genes that encoded DECs and the number of genes that encoded DEGs. (**b**) Diagrams showing genes that encode circRNAs and that lie within the diagram segments on the Venn diagram (**a**). The numbers above the bars indicate the number of circRNAs. (**c**) Hierarchical cluster analysis of genes, encoded DECs and corresponding mRNAs in IR24 versus SH24. Each column represents a comparison group, and each row represents a gene. Green strips represent high relative expressions and pink strips represent low relative expressions, *n* = 3 per group. (**d**,**e**) Changes in expression level of circRNAs and mRNAs in IR24 versus SH24 for the top ten genes that lie within the diagram segments on the Venn diagram. The top ten genes that encoded DECs and that exhibited the greatest fold change in expression, as well as encoded DEGs (**d**) or non-significantly regulated mRNAs (**e**) are shown. The cut-off of RNA expression changes was 1.50. Only those RNAs with *Padj* < 0.05 were selected for analysis. (**f**,**g**) The numbers indicate the circRNAs that are up-, down-, or non-DECs in IR24 versus SH24 and that originated from group 1 (**f**) or group 2 (**g**) genes, respectively.

Previously, using RNA-Seq, we identified 1939 differentially expressed genes that changed their mRNA expression more than 1.5 fold, *Padj* < 0.05 (DEGs) in IR24 versus SH24 [29]. mRNA sequencing data were deposited in the Sequence Read Archive database under accession code SRP148632. Here, further meta-analysis of mRNA sequencing results allowed us to divide the 211 genes that encoded DECs into two groups. In group 1, we included 73 genes that encoded DECs and that overlapped with genes that encoded DEGs in IR24 versus SH24 (Figure 4a, Supplementary Table S3). In group 2, we included 138 genes that encoded DECs, but not differentially expressed mRNAs (non-DEGs) (Figure 4a, Supplementary Table S4). Hierarchical cluster analysis of 211 genes that encoded DECs and corresponding mRNAs in the rat brain subcortex at 24 h after tMCAO is illustrated in Figure 4b.

In both groups, there was a significant number of genes that encoded several DECs as well as non-DECs. Thus, 180 DECs (6 up- and 174 downregulated) and 200 non-DECs were found that originated from the genes of group 1. Furthermore, 215 DECs (12 up- and 203 downregulated) and 492 non-DECs were found that originated from the genes of group 2 (Figure 4c). We found the top 10 genes of group 1 (*Pde10a, Mme, Rgs9, Dgkb, Trank1, Egfem1, Ece1, Mcc, Col4a1,* and *Mvp*) and of group 2 (*Macf1, Ppp1r7, Utrn, Per3, Tnik, Fbxw4, Rps6kc1, Lap3, Myh9l1,* and *Cdyl*) that encoded circRNAs with the greatest fold change of expression. The comparison of the expression level between circRNAs and mRNAs that were encoded by these genes is shown in Figure 4d,e, respectively. It should be noted that the genes of group 1 that encoded mRNAs and circRNAs had predominantly unidirected expression changes (either both mRNA and circRNA were upregulated or both circRNA and mRNAs were downregulated) in IR24 versus SH24. Only the *Egfem1* gene, which provides the synthesis of EGF-like and EMI domain containing 1 protein, encodes mRNAs and circRNAs which had opposite directed expression (upregulated circRNA and downregulated mRNA) in IR24 versus SH24 (Figure 4d). Figure 4f,g shows the number of up-, down- and non-DECs that were encoded by the genes of groups 1 and 2, respectively. It should be noted that we did not find any genes that would encode circRNAs which had opposite directed expression in IR24 versus SH24 (Figure 4f,g).

Analysis of the Gene Ontology database tool allowed the identification of functional diversity between genes that encoded both DECs and DEGs (group 1), as well as genes that encoded only DECs, but non-DEGs (group 2) in the rat brain subcortex after tMCAO model conditions (Supplementary Table S5). The former genes were associated with cyclase (e.g., *Adcy5*) and active transmembrane transporter activity (e.g., *Slc4a4, Atp2b1*), whereas the latter were associated with extracellular matrix binding (e.g., *Spock3*), small molecule binding (e.g., *Prex2, Ric8b*), chromatin binding (e.g., *Mllt3, Ncor1*), channel regulator (e.g., *Wnk2*), guanyl-nucleotide exchange factor (e.g., *Fgd6, Rapgef6, Prex2*), as well as transcription coregulator activity (e.g., *Cdyl, Ncor1*).

### 3.5. Differentially Expressed circRNAs Associated with Neurotransmitter Signaling Pathways at 24 h after tMCAO

Using gProfileR, a pathway enrichment analysis was used to annotate the signaling pathways associated with genes that encoded DECs in the rat brain subcortex in tMCAO model conditions. We found fifteen signaling pathways for genes that encoded DECs in IR24 versus SH24 (Supplementary Table S6). Among them, glutamatergic synapse, dopaminergic synapse, calcium signaling pathway, oxytocin signaling pathway, and long-term potentiation were the top 5 signaling pathways with the maximal count of DECs. Additionally, similar results were obtained using DAVID version 6.8. Thus, based on the DAVID data, upregulated DECs were associated with a glutamatergic synapse, long-term potentiation, dopaminergic synapse, amphetamine addiction, and other pathways (Supplementary Table S7).

**Figure 5:**
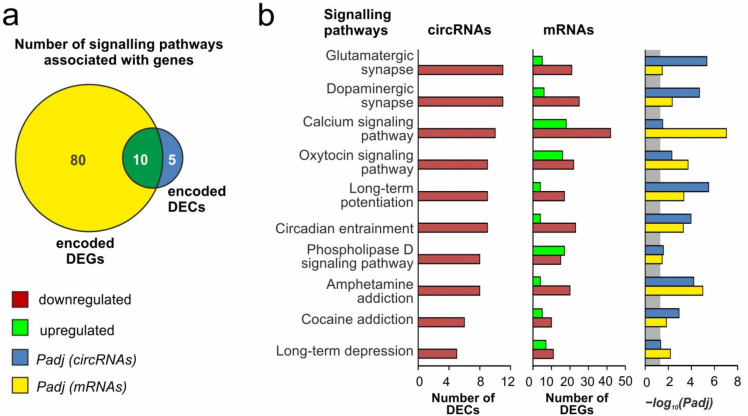
Analysis of the signaling pathways associated with DECs at 24 h after tMCAO. A pathway enrichment analysis of DEGs in pairwise comparisons IR24 vs. SH24 was carried out according to the gProfileR. (**a**) Schematic comparison of the signaling pathways associated with genes that encoded DECs and genes that encoded DEGs obtained in pairwise comparison IR24 vs. SH24 by Venn diagram. The numbers in the diagram segments indicate the number of genes. (**b**) The characteristics of the 10 signaling pathways that lie within the intersection on the Venn diagram (**a**). The number of upregulated and downregulated RNAs, as well as the *Padj*-values, are shown. Only those RNAs and signaling pathways whose *Padj* < 0.05 were selected for analysis. *Padj* ≥ 0.05 are enclosed in the gray background.

Identified signaling pathways are involved predominantly in neurotransmission. Simultaneously, 90 signaling pathways associated with DEGs in IR24 versus SH24 were identified using gProfiler. Furthermore, we revealed that 10 of these pathways were common to both genes that encoded DEGs and to genes that encoded DECs (Figure 5a). It should be noted that identified neurosignaling pathways were predominantly associated with both genes that encoded downregulated circRNAs and genes that encoded downregulated mRNAs in IR24 versus SH24 (Figure 5b).

### 3.6. Analysis of circRNA–miRNA–mRNA Networks Associated with the ‘Glutamatergic Synapse’ Signaling Pathway at 24 h after tMCAO

Using DAVID version 6.8, a Kyoto Encyclopedia of Genes and Genomes (KEGG) pathways annotation was downloaded for all rat genes. Based on these data, only genes that associated with the ‘glutamatergic synapse’ signaling pathway were chosen. Thus, we formed a list of mRNAs and DECs that originated from these genes. In total, 37 DECs and 108 mRNAs, including 26 DEGs, were used for analysis of circRNA-miRNA-mRNA networks.

**Figure 6:**
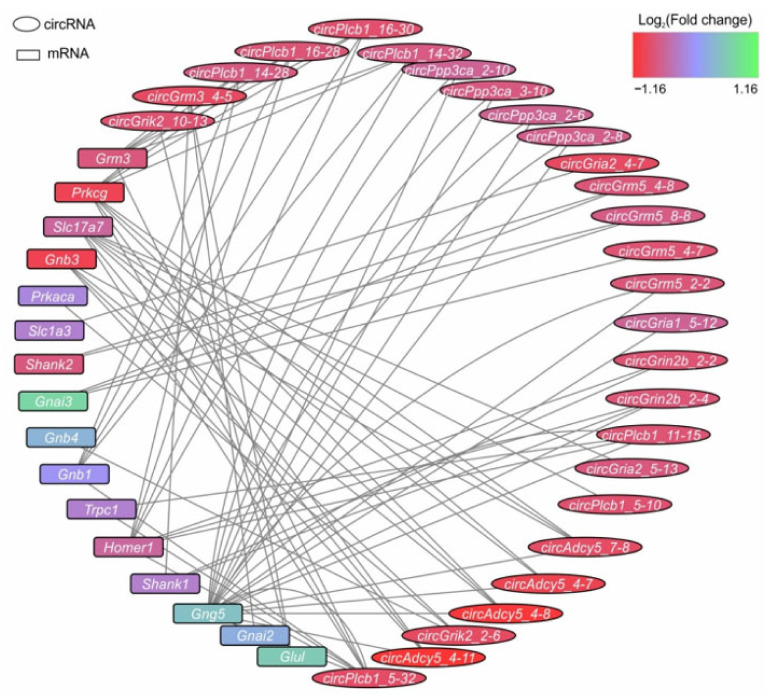
The circRNA–miRNA–mRNA regulatory network for DECs and DEGs, associated with the signaling pathway ‘glutamatergic synapse’. The network was constructed using Cytoscape 3.8.2 software. The nodes are designated the mRNAs or circRNAs. Each line connecting the nodes indicates the interaction of the corresponding mRNAs and circRNAs with common miRNAs. Accordingly, a different number of lines connecting the nodes indicates a different number of miRNAs, for the interaction with which circRNAs and mRNAs compete. The cut-off for circRNA-expression changes was 1.50. Only those circRNAs whose *Padj* < 0.05 were selected for analysis.

To search for miRNA-mRNA pairs, we integrated the results of miRWalk2.0, TargetScan, and miRTarBase. Simultaneously, a search for potential miRNA-circRNA interactions was conducted using the integrated resource CircAtlas. Thus, the circRNA-miRNA-mRNA competitive network for DECs and mRNAs associated with the ‘glutamatergic synapse’ signaling pathway was compiled (Supplementary Table S8). Moreover, Figure 6 shows the network of circRNA-miRNA-mRNA competitive interactions and provides a preliminary insight into the competition between the mRNAs and DECs in IR24 versus SH24 for interaction with miRNAs for glutamate neurosignaling. The nodes are designated the mRNAs or circRNAs in the network. Each line connecting the nodes indicates an interaction of the corresponding mRNAs and circRNAs with common miRNAs. Thus, the number of lines indicates the number of miRNA-mediated competitive interactions between mRNA and circRNA that are linked by these lines.

In the networks, the top five mRNAs that have the greatest number of competitive interactions originated from *Gng5* (20), *Prkcg* (nine), *Homer1* (eight), *Slc17a7* (seven), and *Grm3* (six). It is noteworthy that *Gng5*, *Homer*, and *Slc17a7* mRNAs are non-DEGs, whereas *Prkcg* and *Grm3* are downregulated in IR24 versus SH24 (Supplementary Table S9). The greatest number of miRNA-mediated competitive interactions was found for *circPlcb1_32.5* (8). Other circRNAs (*circPlcb1-15.11*, *circAdcy5_11.4*, *circGrm3-4.5*, *circPlcb1_28.14*, etc.) had four or fewer competitive interactions. Notably, all these circRNAs were downregulated in IR24 versus SH24 (Supplementary Table S10). Furthermore, analysis of circRNA–miRNA–mRNA networks revealed 22 miRNAs that provided the competition between the mRNAs and DECs. The greatest number of competitive interactions was mediated by *rno-let-7i-5p* (11). Other miRNAs (*rno-miR-3075*, *rno-let-7c-5p*, *rno-miR-3542*, *rno-miR-504*, etc.) mediated nine or fewer competitive circRNA-miRNA-mRNA interactions (Supplementary Table S11). Thus, our predicted circRNA-miRNA-mRNA competitive network can persist to verify further functional axes to regulate neurosignaling.

## 4. Discussion

The present study revealed the genome-wide response of the circRNA transcriptome to the damaging effect of IR in tMCAO rat model conditions based on endovascular artery occlusion (90 min) and subsequent reperfusion. This model reflects events that occur in ischemic stroke in humans after treatment with thrombolytic agents [52,53]. The results of clinical studies indicate that thrombolysis is currently one of the most effective and affordable methods for treatment of ischemic stroke [54,55]. MRI results showed that the specific model of tMCAO used in the present study (mode and time of occlusion and reperfusion) allowed the induction of a focal ischemic injury with a predominant subcortical localization. Previously, as a result of pathomorphological studies, we characterized clearly ischemic and peri-infarct regions including both damaged and viable cells [29,30,31,56].

Using RNA-Seq, we identified over 11,000 circRNAs in the subcortical structures of the rat brain under conditions of IR of the brain. Furthermore, a significant change in expression was detected only in 395 circRNAs at 24 h after tMCAO. It is noteworthy that most of these circRNAs were downregulated under IR conditions. Moreover, annotated DECs contained coding exons predominantly. Previously, we identified the human orthologues for a number of rat genes, which expression was changed after ischaemic stroke (*Adora2a, Bcl3, Ccl22, Ccr1, Cd14, Gpr6, Gpr88, Rgs9,* and other genes) [57]. Those results gave us the basis for examination of genetic variants revealed from a rat model of brain ischemia in patients with ischemic stroke. Here, it was found that at least 93 DECs may have orthologous human circRNAs that also may serve a launch point for consideration in patients. We have shown that many genes encoded both several DECs and several circRNAs, whose differential expression by the RNA-Seq method was not significant. Using a microarray of ischemic penumbral cortex from mice, Mehta et al. (2017) revealed that under ischemia conditions at 6, 12, and 24 h after tMCAO, the expression of 239, 41, and 53 circRNAs, respectively, was changed [19]. Interestingly, we found no genes that encoded DECs or that overlapped with the results of Mehta et al. (2017) at 24 h after tMCAO. However, there were 17 genes (*Cdyl, Ttc3, Tnik, Ylpm1, Erc2, Grin2b, Nrd1, Cdk17, Taok1, Nsd1, Vps13b, Akap11, Fbxw4, Fat3, Ank3, Zfp644,* and *Nrxn3*) that also encoded DECs in the ischemic cortex in mice at 6 or 12 h after tMCAO. Thereby, a small number of overlapping circRNAs were revealed comparing different modes of ischemic damage. This result may obviously be associated with the use of various species of animals and different methods of expression analysis. Simultaneously, it may also reflect significant differences in the mechanisms of action of circRNAs in different parts of the brain that exist in different conditions of ischemic damage. It is possible that there is an active spatial–temporal regulation of the expression of circRNAs in the brain under conditions of cerebral ischemia. However, it should be noted that almost all overlapped circRNAs decreased their expression a day after tMCAO model conditions. It is likely that these circRNAs can serve as a novel biomarker of, and therapeutic target for, stroke.

Previously, we used high-throughput RNA sequencing to analyze genome-wide mRNA expression in a rat model of tMCAO [29]. We identified 1939 genes encoding differentially expressed mRNA (DEGs) under IR conditions after tMCAO at 24 h [29]. A DAVID version 6.8 enrichment analysis showed that dozens of functional annotations were associated with DEGs under IR conditions. Thus, the activation of a large number of genes was associated with inflammatory, immune, stress and other processes, whereas massive downregulation of the mRNA levels of genes involved in the functioning of neurotransmitter systems was recorded [29]. Those results allowed us to compare differential expression of circRNAs and mRNAs. In the present study, we discovered that DECs originated from a much smaller number of genes, namely 211, and that around one-third of them originated from genes whose mRNA levels also changed at 24 h after tMCAO (Figure 4a, Supplementary Table S2). Other DECs originated from genes whose mRNA levels were not regulated under tMCAO conditions (Figure 4a, Supplementary Table S3). Both the genes that encoded DECs and DEGs (group 1), and those that encoded DECs but non-DEGs (group 2) had predominantly neurotransmitter functional annotations, but differed according to some specific subtype of common molecular function of their proteins (Supplementary Table S4). The former were genes associated with cyclase and active transmembrane transporter activity, whereas the latter were associated with extra-cellular matrix binding, small molecule binding, chromatin binding, channel regulation, guanyl nucleotide exchange factor, and transcription coregulator activity. Recently, a few studies comparing the expression profiles of mRNAs and long non-coding RNAs (lncRNAs), including circRNAs, have been published. Specifically, profiling analysis of circRNAs and mRNAs in human temporal lobe epilepsy with hippocampal sclerosis has been conducted [44]. Furthermore, microarray and functional enrichment analysis of circRNAs and mRNAs in a mouse model of intestinal IR injury with and without ischemic post-conditioning was performed [58]. Here, our results may indicate the specificity of the formation and degradation of circRNAs and mRNAs during cerebral IR, as well as the specific mechanisms of functioning of various linear and circular RNAs, which appear to be related to the uniqueness of their structures.

In the present study, functional enrichment analysis of genes that encoded DECs revealed a number of signaling pathways, which mainly determined the transmission of signals in the central nervous system. It is important to note that most of them overlapped with the signaling pathways associated with genes that encoded DEGs (Figure 5a, Supplementary Tables S5 and S6). In particular, at 24 h after tMCAO, we found that genes that encoded downregulated mRNAs and downregulated circRNAs were involved in the regulation of the functioning of the glutamatergic synapse, dopaminergic synapse, calcium signaling pathway, and other functions (Figure 5b). It is noteworthy that massive downregulation of circRNAs after tMCAO cannot be associated with a decrease in the number of survival cells in the necrotic zone alone, because we previously found activation of a large number of genes associated with inflammation, apoptosis, immune, stress, and other responses under tMCAO conditions [29]. According to some studies, the expression of circRNAs under various conditions in models of ischemia may be associated with the metabolic pathways of apoptosis and immune response [18], with metabolic processes, cell communication, and binding to proteins, ions, and nucleic acids [19], as well as with signaling pathways regulating the processes of cell survival and death [20]. Previously, we showed that experimental cerebral ischemia affects the expression of circRNAs of genes for metabotropic glutamate receptors mGluR3 and mGluR5 in the rat brain [59]. Undoubtedly, genome-wide transcriptome profiling has expanded our current knowledge about the neurotransmission regulation in the rat brain after IR with circRNA action.

At present, considerable attention is being paid to the study of the function of circRNAs as miRNA sponges. CircRNAs acting as competitive endogenous RNAs (ceRNAs) compete with mRNAs for binding to miRNAs and diminish the effect of miRNAs on transcriptional and post-transcriptional levels of regulation of gene expression [60,61]. The functions of several circRNAs as miRNA sponges have been investigated in various pathologies. In particular, the role of circRNA CIRs-7 in preventing models of neuropsychiatric disorders in mice, associated with its functioning as a ceRNA, was recently established [61]. In addition, in Alzheimer disease [62], Parkinson disease [63], and various types of cancer [64,65,66], investigators found that circRNA–miRNA–mRNA competition may be associated with pathogenesis regulation. Under various conditions in models of ischemia, possible interactions between circRNAs and miRNAs, which can provide potential information to reveal the mechanisms of brain damage in ischemic stroke, were predicted and circRNA–miRNA–mRNA axes/networks were constructed [18,19,20,21,25,26,27,28]. In the present study, we predicted a circRNA–miRNA–mRNA competitive network based on the RNAs associated with the ‘glutamatergic synapse’ signaling pathway (Figure 6). This signaling pathway was highly associated with the genome-wide expression profile of both mRNAs and circRNAs at 24 h after tMCAO. Moreover, such RNAs were predominantly downregulated under IR conditions. Previously, regulation of the glutamatergic system was shown to be associated with circRNA functioning. circRNA for *Grm1* promoted pulmonary artery smooth muscle cell proliferation and migration [67]. CircRNA for *Gria1* showed an age-related increase in male macaque brain and regulates synaptic plasticity and synaptogenesis [68]. Additionally, a number of circRNA–miRNA–mRNA axes are associated with glutamate genes in cancer [69,70,71]. Our present data show that one mRNA may compete with many more than one circRNA, and vice versa. It is noteworthy that the multiple (pleiotropic) action of circRNAs may be one of the remarkable regulatory properties of these molecules. As a result, many mRNAs can be protected from miRNA-induced silencing. The functioning of *circHECTD1* is an example of such pleiotropic action of circRNAs. The role of this molecule in at least three different regulatory axes (e.g., *circHECTD1–miR-27a-3p–FSTL1* [72], *circHECTD1–miR-133b–TRAF3* [26], and *circHECTD1–miR-142–TIPARP* [23]) has been shown experimentally. According to our data, under IR conditions at 24 h after tMCAO, the greatest number of circRNA–miRNA–mRNA competitive interactions was for *Gng5* mRNA (20) and *circPlcb1-32.5* (8). Thus, it can be assumed that such circRNAs and mRNAs may be the nodes of the most active regulation of the neurotransmission genetic response under conditions of brain IR. It is notable that the gene for phospholipase C beta 1 (*Plcb1*) is the host for *circPlcb1-32.5* and is included in several circRNA-miRNA-mRNA regulatory axes [73,74]. *Gng5* encodes G-protein subunit γ-5, which is required for the GTPase activity, to replace guanosine diphosphate (GDP) by guanosine triphosphate (GTP), and for G-protein–effector interaction. Furthermore, miRNAs were also shown to be involved in regulating *Gng5* function. Thus, *miR-675-3p* regulates IL-1β-stimulated human chondrocyte apoptosis and cartilage degradation by targeting *Gng5* [75]. We showed that *Gng5* mRNA level was almost unchanged at 24 h after tMCAO [29]. It is therefore possible that multiple circRNA–miRNA–mRNA competitive interactions provide greater resistance to *Gng5* mRNA under IR conditions. Simultaneously, we found that the level of expression of most circRNAs was decreased at 24 h after tMCAO, and such circRNAs may actively compete with the mRNAs. Moreover, such circRNAs accompany a decrease of the mRNA levels of genes involved in the functioning of neurotransmitter signaling pathways. It is possible that a decrease in circRNA level leads to a prevailing downregulation of mRNAs for neurotransmitter genes, because the number of molecules capable of protecting mRNAs from miRNA-induced repression is substantially diminished.

A limitation of our study is the use of computational tools alone to identify circRNA–miRNA–mRNA networks. A combination of experimental and bioinformatics techniques to establish the active circRNA–miRNA–mRNA axes would further elucidate the mechanisms of neurosignaling associated with IR in various brain structures.

## 5. Conclusions

Our study reveals that most differentially expressed circRNAs are downregulated in response to IR conditions of the rat brain. Functional annotation revealed that these circRNAs are predominantly associated with neurotransmission cell systems. Bioinformatic analysis predicted several potential circRNA-miRNA-mRNA regulatory nodes, which may determine molecular mechanisms of neurotransmission signaling pathway regulation in brain cells under IR conditions. Subsequent verification of circRNA-miRNA-mRNA interactions will be an important step in determining strategies for achieving a neuroprotective effect in the conditions of cerebral ischemia.

## Data Availability

Publicly available datasets (PRJNA523319 (SAMN10970974-SAMN10970979, https://www.ncbi.nlm.nih.gov/sra/PRJNA523319, accessed on 18 Febrary 2020) [50]; SRP148632, (PRJNA472446 (SAMN09235828-SAMN09235839, https://www.ncbi.nlm.nih.gov/Traces/study/?acc=SRP148632, accessed on 6 Febrary 2019) [76] and GenBank accession number MK520929 [51]) were analyzed in this study.

## Supplementary Materials

The following are available online at https://www.mdpi.com/article/10.3390/genes12121870/s1, Figure S1: The number of genes and circRNAs that were encoded by them, Table S1: The characterization of the primers for RT-PCR, Table S2: A search for orthologous rat DECs in human (a pilot study), Table S3: Genes that encoded both differentially expressed circRNA and mRNA in IR24 versus SH24, Table S4: Genes that encoded DECs, but non-DEGs in IR24 versus SH24, Table S5: Analysis of the molecular functions associated with genes from group 1 and group 2 using the PANTHER tool, Table S6: Analysis of the signalling pathways associated with DECs and DEGs at 24 h after tMCAO using gProfileR, Table S7: Analysis of the signalling pathways associated with DECs and DEGs at 24 h after tMCAO using DAVID version 6.8, Table S8: The characteristics of circRNA-microRNA-mRNA regulatory network for DECs and mRNAs associated with the ‘glutamatergic synapse’ signalling pathway, Table S9: The number of competitive interactions for mRNAs, Table S10: The number of competitive interactions for circRNAs, Table S11: The number of competitive interactions that were mediated by miRNAs.
