# Supplementary material for: Genome-Wide RNA-Sequencing Reveals Massive Circular RNA Expression Changes of the Neurotransmission Genes in the Rat Brain after Ischemia–Reperfusion
Source: Genes (Basel). 2021 Nov 24;12(12):1870. doi: 10.3390/genes12121870 (PMC8701796; doi:10.3390/genes12121870)
Supplement: Supplementary file 1 [file genes-12-01870-s001.zip › genes-1456631-supplementary/Supplementary Figure S1.pptx]

## Slide 1
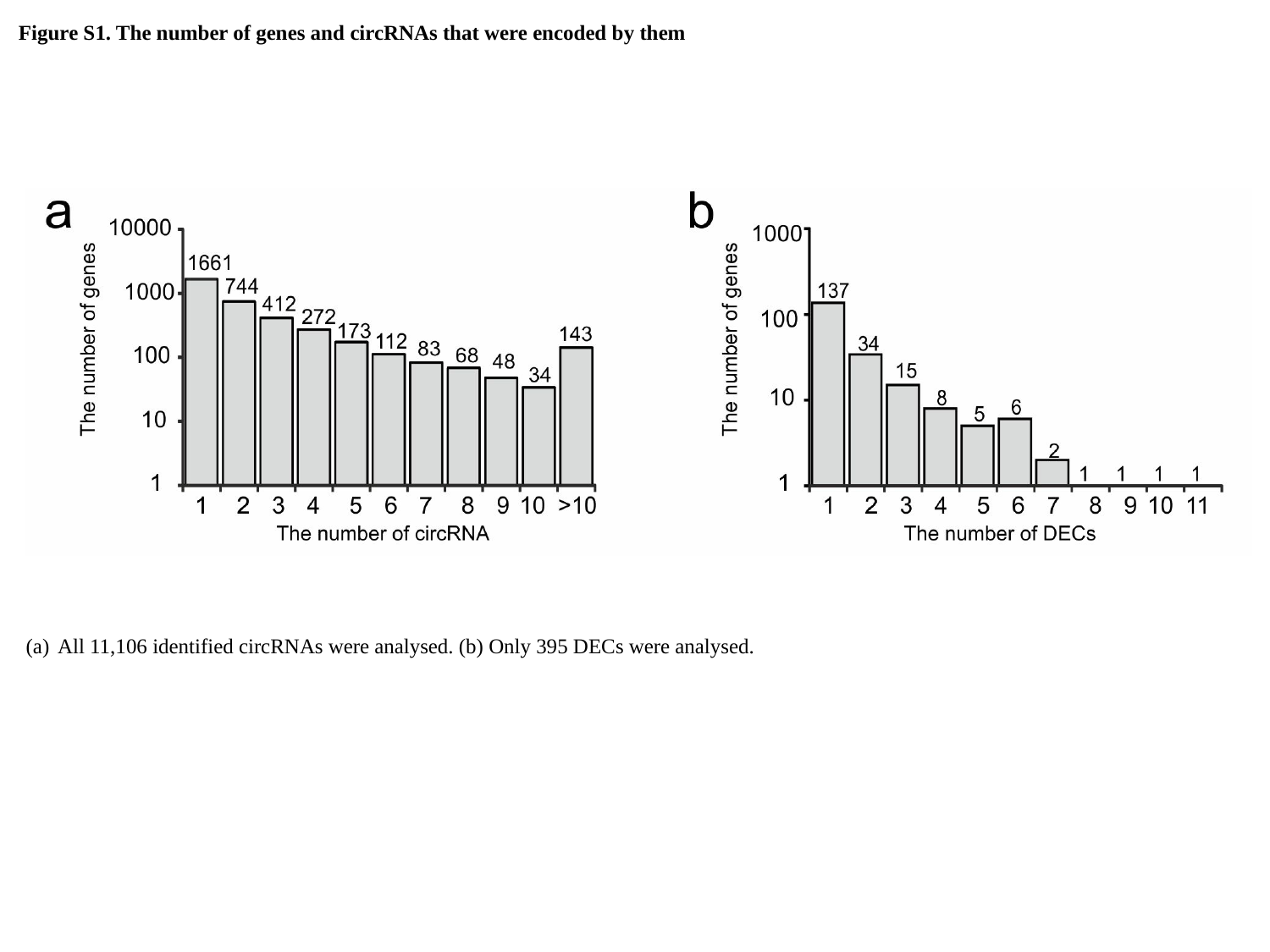

Figure S1. The number of genes and circRNAs that were encoded by them
All 11,106 identified circRNAs were analysed. (b) Only 395 DECs were analysed.
